# Extended Perspective Shift and Discourse Economy in Language Processing

**DOI:** 10.3389/fpsyg.2021.613357

**Published:** 2021-03-31

**Authors:** Jesse A. Harris

**Affiliations:** Department of Linguistics, University of California, Los Angeles, Los Angeles, CA, United States

**Keywords:** perspective taking, free indirect discourse, tense, discourse processing, language processing

## Abstract

Research spanning linguistics, psychology, and philosophy suggests that speakers and hearers are finely attuned to perspectives and viewpoints that are not their own, even though perspectival information is not encoded directly in the morphosyntax of languages like English. While some terms seem to require a perspective or a judge for interpretation (e.g., epithets, evaluative adjectives, locational PPs, etc.), perspective may also be determined on the basis of subtle information spanning multiple sentences, especially in vivid styles of narrative reporting. In this paper, I develop an account of the cues that are involved in evaluating and maintaining non-speaker perspectives, and present an economy-based discourse processing model of perspective that embodies two core principles. First, perspectives are subject to a “speaker-default,” but may shift to a non-speaker perspective if sufficient contextual cues are provided. Second, the processor follows the path of least resistance to maintaining perspective, opting to maintain the current perspective across sentences as long as the shifted perspective continues to be coherent. The predictions of the model are tested in a series of offline and online studies, manipulating the form of an attitude report and the tense of the sentence that follows. Implications for processing perspective and viewpoint in speech and narrative forms are explored.

## 1. Introduction

Language users are clearly sensitive to perspectives other than their own. And yet, there appears to be no uniform or determinate way to convey point of view, even though many lexical items, e.g., *beautiful* or *nearby*, seem to require that a viewpoint be assessed for interpretation (e.g., Mitchell, 1986; Partee, 1989; Lasersohn, 2005; Stephenson, 2007, among many others in linguistics and philosophy). Perspective has been described in various ways: as an “origo” (Bühler, 1934), an “empathic identification” (Kuno and Kaburaki, 1977), a “judge” (Lasersohn, 2005), an “evaluator” (Patel-Grosz, 2012), or, as we do here, a “perspectival center” (Harris, 2012; Patel-Grosz, 2012; Hinterwimmer, 2017). Still, little is known about how a perspectival center is calculated by the human language processing system, and we are, at present, left with a wealth of open foundational questions: Given the myriad of potentially relevant information sources that signal perspective, how does the processor determine which perspective is at play? What types of cues signal a perspective shift? How do these cues interact? Is perspective shift costly to maintain over longer stretches of discourse or text?

Most experimental research takes as its starting point the assumption that, in non-narrative genres, a presumptive pragmatic default favors the speaker's perspective, which then interacts with presentational, surface cues to shift to a non-speaker center (Smith, 2003, 2009; Harris, 2012), even if the mechanisms motivating these shifts differ dramatically. Many lexical and contextual cues have been explored experimentally: epithets (Harris and Potts, 2009, 2011; Kaiser, 2015) or other expressives, like *damn* (Frazier et al., 2015), verbs of saying (Harris and Potts, 2009), evaluative adjectives (Kaiser and Lee, 2018; Kaiser, to appear), as well as subtle prosodic and non-verbal modulations (Harris and Potts, 2011).

This project turns to how perspective shift is achieved in vivid narration style, in which the perspectival center shifts to a third-person character. Utilizing the perspective shifting tendencies of narrative parenthetical reports, as in *It was a time of utter chaos, lamented Mary*, this paper explores how the tense of a following continuation influences whether the perspective stays shifted with the third-person or reverts back to the speaker. To this end, I present a series of offline and online experiments that manipulate the report type of an utterance and the tense of the sentence that follows. The results confirm that, compared to standard indirect styles of reporting, narrative parenthetical reports provide a strong cue for an alternate perspectival center (Reinhart, 1975, 1983). Further, a shifted perspective is more likely to extend to the following sentence when it is presented in Present tense, placing the reported event within a single, continuous “contextual now.” I argue that the results support an economy-based model of perspective processing, in which the discourse processor discourages perspective shifting generally, but maintains a shifted perspective when possible for continuity.

## 2. Perspective and Perspective Shift

Intuitively, a perspective relates to a particular attitude, opinion, or belief regarding an individual or situation. It is roughly synonymous with *viewpoint* and *point of view*. In this sense, a perspective then is just a body of perceptions, commitments, and information, broadly construed, that hold for an attitude holder in a particular situation. Following Harris and Potts (2009), a constituent or clause C is said to be speaker-oriented if the speaker expresses a commitment to the content of C in using it; otherwise, C is said to be non-speaker-oriented, and expresses, perhaps by proxy of the speaker, the commitments and perspectives of another individual in the discourse.^1^ I will use the term perspectival center as a shorthand for how the views and commitments of a relevant attitude holder are represented within the discourse. The term is intended to be compatible with multiple formalizations of linguistic subjectivity.

Some lexical items seem to require a perspectival center for interpretation. For example, certain locational predicates like *local* or *nearby* is evaluated with respect to a particular location (Mitchell, 1986; Partee, 1989; Recanati, 2011). Expressives and other evaluative terms, like predicates of personal taste, likewise convey a particular viewpoint (e.g., Lasersohn, 2005; Potts's, 2005, among many others). By using an epithet like *that jerk Bill*, the speaker conveys that she (or another attitude holder) stands in a particular, usually negative, emotional relationship with the referent, Bill. Similarly, the use of *tasty* in *that dish is tasty* indicates that an attitude holder takes a positive gustatory stance with respect to the dish under discussion.

Since expressives typically convey the experience or opinion of an individual,^2^ they are sometimes said to directly update the context with the relevant attitude (Potts, 2007; Barker et al., 2011). Expressive content is thus notoriously challenging to deny. In Potts's (2005) examples (1-2), an epithet like *that bastard* communicates a certain degree of speaker commitment to the attitude conveyed by the epithet. To then immediately renounce or deny that commitment is strikingly incoherent (1). Expressive judgments also seem impervious to standard forms of denial. While it's easy enough for Speaker A to assert another opinion (2a), a direct denial of Speaker A's attitudinal stance (2b) is inappropriate (unless perhaps to convey the implication that A's attitude is insincere or fleeting). Nonetheless, the veridicality of A's attitude itself can be called into question, even if it has not been denied directly (2c).


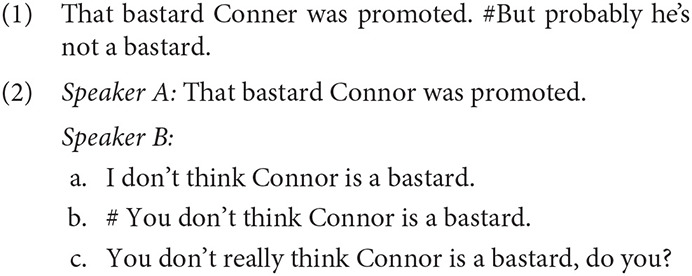


As predicates of personal taste convey personal or subjective experience, they are subject to what has sometimes been called “faultless disagreement,” in which opposing opinions regarding personal taste might each be true (e.g., Kölbel, 2004). Intuitively, Abe's claim in (3) is false if it generically extends to others, including Betty, but true enough if it is merely limited to his own preferences.


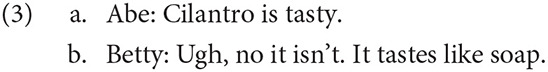


The above properties have fueled the claim that expressives and related terms are inherently speaker-oriented (Cruse, 1986; Kaplan, 1999; Potts's, 2005). An intuitive explanation is that one's feelings and emotions are one's own, and that other discourse participants simply lack the appropriate first-hand experience to warrant a denial (e.g., Willer and Kennedy, to appear).

And yet, many cases of non-speaker perspective shift have been documented in the literature. Following the intuitions of Amaral et al. (2007), Wang et al. (2005), and others, Harris and Potts (2009) showed experimentally that such terms could be understood as reflecting a non-speaker perspective, provided there were sufficient clues in the context to warrant a shift in perspective (see also Harris, 2009, 2012; Kaiser, 2015). Their results found that changing the valence of a single word (e.g., *high* to *low*) in context sentences like (4) modulated whether the rate at which subjects attributed an epithet like *the bastard* in the target sentence (5) to the attitude holder (Sheila) or the speaker. In (4A), giving someone a high grade is unlikely to result in considering the professor a jerk, and so the non-speaker interpretation remains unlikely. However, receiving a low grade would plausibly result in a negative assessment, increased non-speaker interpretations of the target (4B).


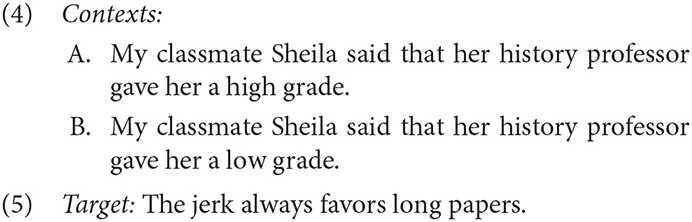


Harris and Potts (2009) proposed that such terms normally default to a speaker-oriented interpretation because shifts in perspective constitute a risky communicative strategy (see also Lasersohn, 2007; Potts, 2007). Such terms do not merely describe a situation, but also evaluate it against a particular perspective or point of view. Without a morphosyntactic or highly conventionalized means to convey non-speaker commitment, speakers run the risk of the content “leaking” from a reportive context. Thus, speakers tend to either avoid non-speaker oriented uses of expressives or limit such cases to rich contexts where their intentions are likely to be recoverable.

Predicates of personal taste can also be understood from a non-speaker perspective under similar circumstances. Stephenson (2007) provided examples in which *tasty* is understood as non-speaker oriented if another perspective is highly salient (6) or embedded under an epistemic modal (7).


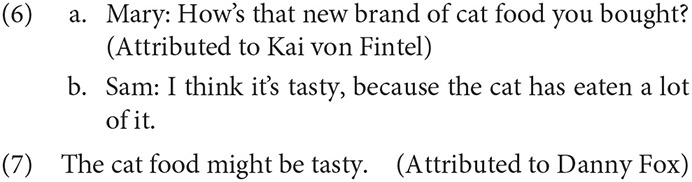


In questions, predicates of personal taste are readily associated with the addressee, participating in “interrogative flip” (e.g., Speas and Tenny, 2003; Tenny, 2006). The examples below are striking in that the speaker's dissenting judgment is clearly known (8) or can be easily inferred (9).


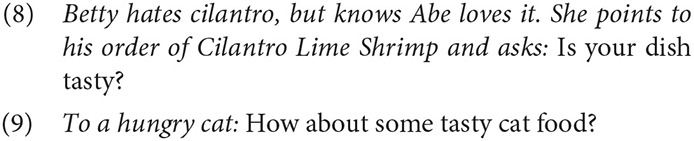


In these cases, it's clear that the speakers are not interested in whether or not they would enjoy the item; the query is entirely oriented toward the perspective and personal tastes of the addressee. Other uses are possible, of course. For example, uttered in a context where Betty does likes cilantro but is unsure about shrimp, (8) could be easily understood as a query into the dish's quality, information that she could use to determine whether she might like it herself.

In an experimental study, Kaiser (to appear) found that the perspective associated with a predicate of personal taste (*disgusting*) was influenced by whether a character was presented as having a direct sensory experience. Experiencer predicates with olfactory (10b), gustatory (10c), and visual sensation (10c) were compared against a non-experiencer baseline (10a).


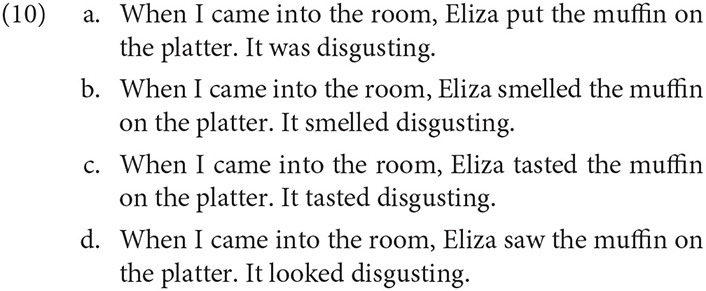


Subjects selected forced-choice answers to questions like *Whose opinion is it that the muffin looked/smelled/tasted/was disgusting?* to indicate whose experience (*the narrator's* or *Eliza's*) was represented by the subjective adjective (*disgusting*). While all experiencer predicates increased the rate of perspective shift, predicates describing taste and smell experiences tended to shift perspective more often (~80–85%) than visual experience (~50%), compared to the baseline (<25%). Presumably, visual experience can, in a sense, be shared between the speaker and another agent, making it less clearly subjective. The results point to the role that experience and evidential warrant play in shifting the perspectival center away from the speaker default. The result aligns with Hinterwimmer (2019) account, in which experiencer predicates generate particularly salient events of the represented experience, thereby providing highly salient anchors for perspective shift.

Even if the evaluation of a term tends to default to the speaker's point of view, shifts in perspective are ubiquitous in text and speech. Single sentence examples can obscure these intuitions, but extended perspective shifts are readily found in many forms of narrative, where events are often presented within a character's perspective. For example, narratives often convey a text through the lens, as it were, of a character telling the story from her experience (see for example Banfield, 1982, Fludernik, 1993, or Emmott, 1999 for review).

The following passage (11) from Henning Mankell's (2009) *The Troubled Man* illustrates the variety of perspectives that may come into play in narrative discourse. The sentences are segmented using the numbered | symbol, marking plausibly non-speaker-oriented passages with an asterisk * superscript.


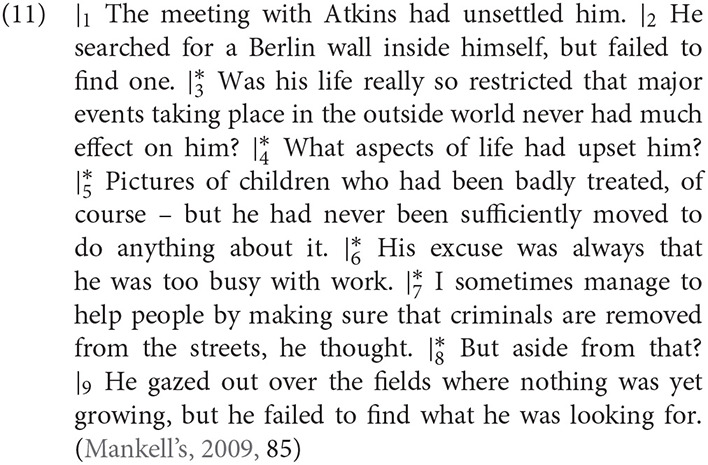


In sentences 3–8, the text contains a variety forms, pronouns, tenses, and non-standard reporting styles, giving rise to the inference that the perspective has shifted from the narrator to the main character's internal thoughts and experience. Although many authors have identified the same kinds of perspective shifting devices in non-reportive narrative styles (e.g., Banfield, 1982; Fludernik, 1993; Smith, 2003), the cues remain somewhat heterogeneous.

The remainder of the paper is organized as follows. A novel taxonomy of perspective shifting cues is proposed in section 2.1, where two natural classes are introduced: cues involved in *evaluating* a perspective, and cues responsible for *maintaining* a shifted perspective. The key features of an economy-based discourse processing model are presented in section 2.2. Then, sections 3–5 present the results of three experimental studies probing the prediction of this model. Finally, section 6 concludes.

### 2.1. Perspective Taking

This paper centers on two theoretical issues central to perspective taking in language. The first addresses what features of a context *initiate* perspective shift. While this is largely an empirical question, any term which is perspectively rich or conventionally associated with a non-speaker perspective could in principle be a candidate. The second issue addresses what factors *maintain* a perspective shift, once shifted. On the assumption that perspective shift is possible, albeit difficult, to maintain across larger stretches of discourse, it is reasonable to expect that a non-speaker perspective is facilitated by cues that reference the speech, thought, or attitudes of the perspective to which the context has shifted.

Prior literature has identified a host of seemingly heterogenous forms contributing to linguistic subjectivity, exemplified by the partial list in (12) from Smith (2003, p. 176):


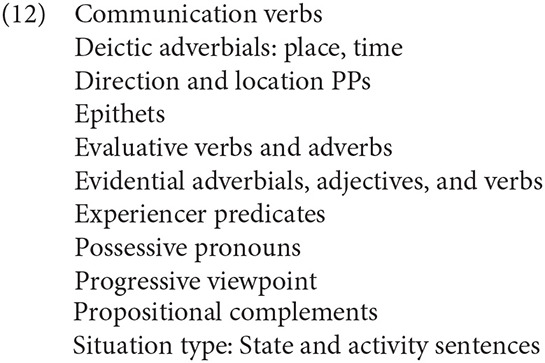


As discussed below, other indicators of perspective, such as certain tense-aspect combinations, interjections, fragments, and questions, may be added to the list.

**Figure 1:**
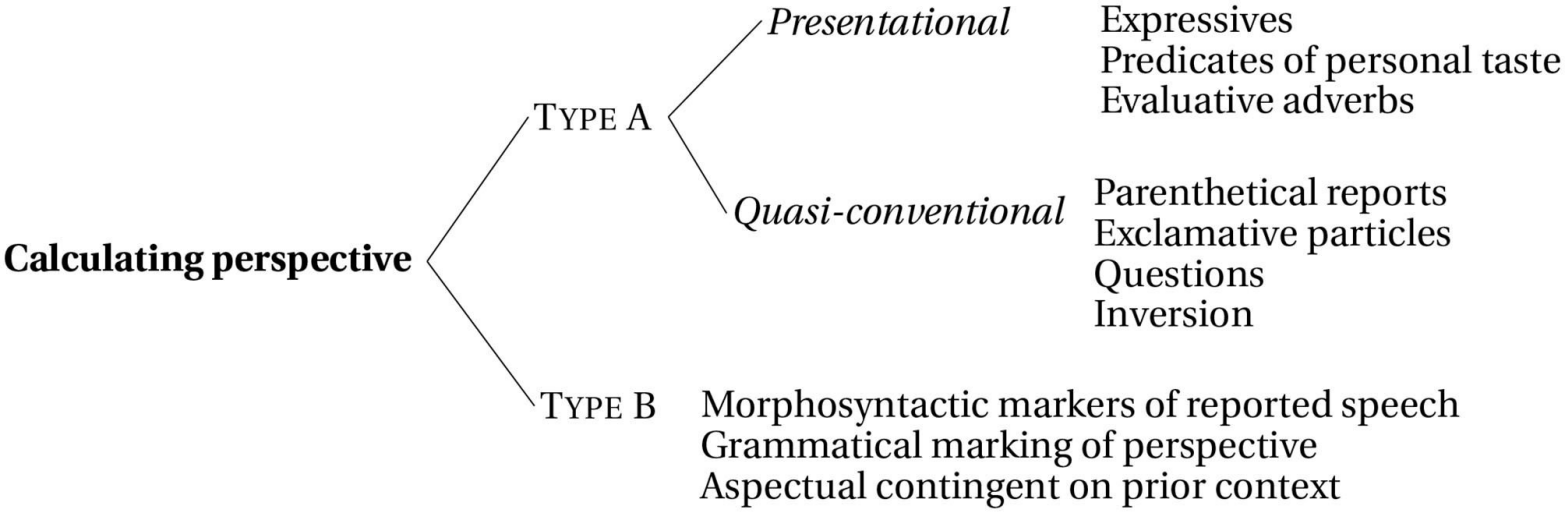
Typology of cues used to calculate perspective. Cues are intended to be representative and not exhaustive.

In the following sections, I propose that perspectival cues may be roughly subdivided into at least two natural classes of cues to perspective by their primary role of establishing (Type A) or maintaining (Type B) a non-speaker perspective. A basic and incomplete sketch of the proposed typology is provided below, summarized in Figure 1. I argue that such cues not only work together, conspiring to strengthen the chance of a perspective shift, but also have distinct roles in interpretation (see also Wiebe, 1994, for the idea that cues to perspective differ in strength). The two types are briefly sketched below. Special attention is given to narrative parenthetical reports and tense, factors that were manipulated in the experiments presented below.

#### 2.1.1. Type A: Evaluating Perspective

The Type A class consists of elements which are semantically or conventional associated with a perspectival center for interpretation. This type may be further divided into two subcategories: presentational and partially or quasi-conventional cues. Type A presentational cues contain an evaluative or representational component for which a judge or perspective is required for interpretation, as in epithets and predicates of personal taste. Additional examples plausibly include communication verbs, deictic adverbs, and evaluative verbs and adverbs like *amazingly*. Examples of Type A quasi-conventional cues consist of cases of root clause phenomena (e.g., Emonds, 1970), including narrative parenthetical reports (13a), exclamative particles (13b), inversion (13c), and questions (13d), among others.^3^


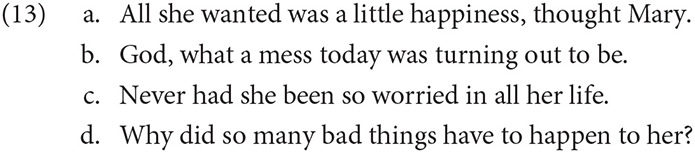


These cues have been studied extensively in the literature on narrative discourse, particularly in works dealing with the phenomena of represented speech and consciousness (e.g., Banfield, 1982; Fludernik, 1993). It is easy to read (13) as a passage conveying the perspective of the parenthetical subject, Mary, rather than the viewpoint of a narrator or speaker. Indeed, Parenthetical reports (PRs) like (13a) are often used to illustrate narrative shifts in perspective. We briefly digress to describe how PRs differ from Standard reports (SRs), and why the former have been implicated in perspective shift.

Although early accounts (Emonds, 1970; Ross, 1970) attempted to derive PRs from SRs, there is strong evidence that they are distinguished both syntactically and interpretively. As Banfield (1982) observed, PRs and SRs have syntactically distinct distributions. For example, PRs generally do not allow complementizers (14), but do permit speaker-oriented adverbs (15), imperatives (16), and direct questions (17) in the reported content.


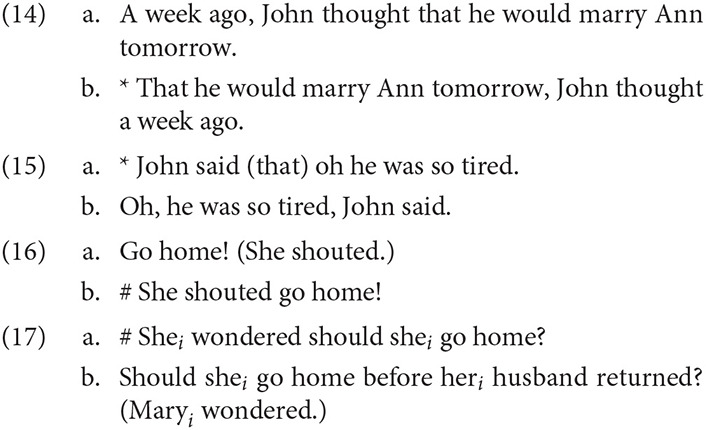


Regarding interpretation, Reinhart (1983) noted that PRs lack an ambiguity that SRs exhibit. The SR in (18) may be interpreted as either a transparent belief, in which the term “his mother” is interpreted *de re*, and is not attributed to Oedipus, or an opaque belief, in which the contents of the belief are interpreted *de dicto*.


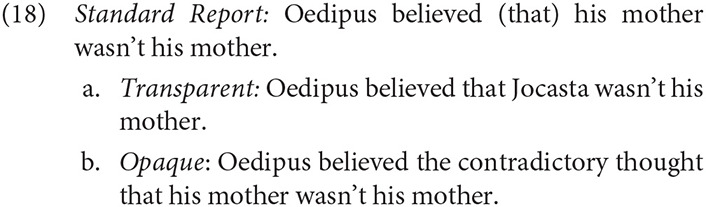


However, PRs lack the transparent reading, and can be interpreted only in the opaque, and in this case a contradictory, *de dicto* sense.


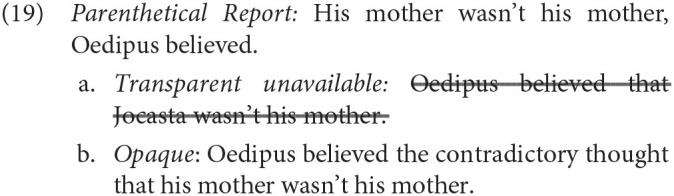


A second, and related, piece of evidence is that a PR represents the parenthetical subject's words or inner speech more directly. Thus, interjections of an opposing perspective from another attitude holder are perceived as infelicitous. Oedipus' thought in (20) may be conveyed as a PR (20a), but only if the report is consistent with Oedipus' actual thought. Interjecting that thought with a speaker-oriented appositive like “his mother in reality” in (20b) is not part of the content of the report, and renders the sentence semantically incoherent.


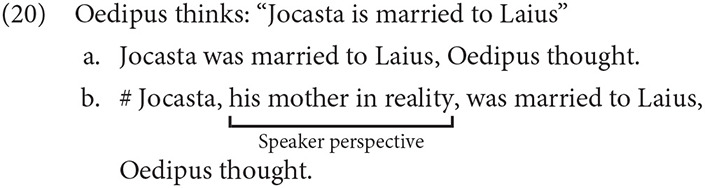


In her seminal analysis of free indirect discourse (FID), Banfield (1982) provided the category of “represented speech and thought,” distinct from directly quoted (DQ) or reported speech. These reports are syntactically realized by an Expressive node consisting of SELF and NOW values, which represent the attitude holder and moment of consciousness, respectively. These values favor the speaker and present, but may be shifted to take on values corresponding to another attitude holder. Many subsequent analyses proposed that expressions are semantically evaluated against two potentially different contexts: one anchored to the utterance situation, the other to the thought or perception situation (e.g., Schlenker, 2004; Sharvit, 2008; Stokke, 2013; Eckardt, 2014; Hinterwimmer, 2017; Abrusán, 2020, among others). Although these accounts differ a good deal in implementation, the two contexts diverge in cases of free indirect discourse and other kinds of perspective shift. This view nicely captures the fact some context-dependent expressions are sensitive to different contexts in FID. While tense and first person pronouns are understood from the speaker's perspective, as in direct discourse, third person pronouns and adverbials optionally reflect the perspective of the represented attitude holder. Crucially, the past tense in FID is understood as past to the speaker, while nonetheless representing the character's contextual here and now (Banfield, 1982; Fludernik, 1993). Example (21) from Sharvit (2008) illustrates the properties that FID shares with DQ (underlined) and the properties it shares with standard indirect reports (**in bold**).


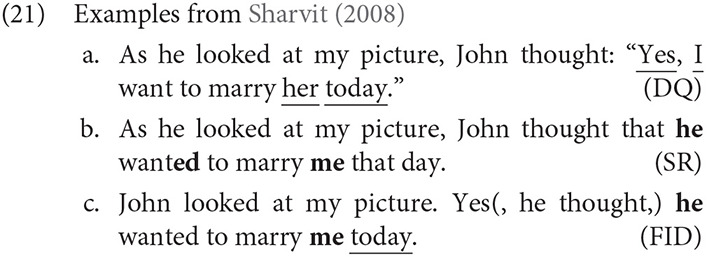


Interestingly, the speech or thought that is represented in FID reports shares some features with DQ, in that various adverbs, like the speaker-oriented adverb *yes* or the temporal adverb *today* in (21a,c), portray the point of view of the reported subject, rather than the speaker, or narrator in the text. As such, it may be tempting to treat FID as a special case of DQ. However, there are important differences between FID and DQ, as well: in FID, a third person pronoun may refer to the subject of the reporting clause, which is *inconsistent* with DQ, but consistent with Standard reports (21b). As noted by Sharvit (2008), there is some disagreement about how first and second person pronouns pattern in FID, but at least in the case above, the first person pronoun appears to refer to the speaker, rather than the attitude holder. Consequently, the extent to which such reports can be treated as an instance of mixed quotation (e.g., Maier, 2015, 2017) remains a matter of some debate (Reboul et al., 2016, for review).

It is important to note that Reinhart (1983) distinguished between two subtypes of parenthetical report: the Narrative parenthetical and the Discourse parenthetical. Only Narrative parentheticals unambiguously represent the point of view of the parenthetical subject. The two types can also be distinguished syntactically. For example, Narrative PRs can postpose the subject to a position after the verb (*said John*), but Discourse PRs cannot. Narrative PRs allow backwards anaphora (22), but do not permit negation (23), in contrast with Discourse PRs.


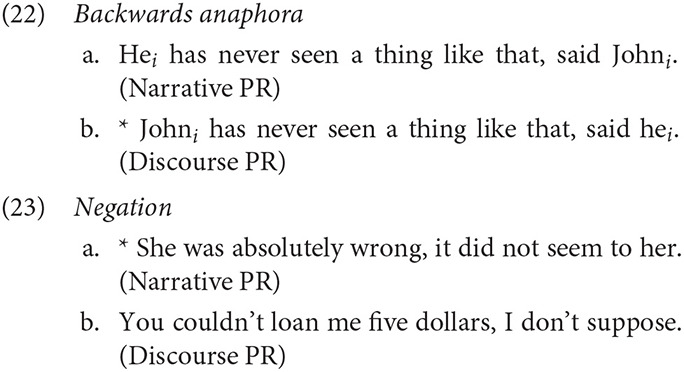


To ensure that the PRs are truly associated with perspective shift in the experiments below, only cases with postposed subjects were used, forcing a Narrative PR reading.

The discussion so far has concentrated on the cues that signal a shift in perspective. However, shifts in perspective may *extend* across several sentences in vivid styles of reporting like FID. The next sections turns to the linguistic elements that are involved in maintaining a non-speaker perspective.

#### 2.1.2. Type B: Maintaining Perspective

In some languages, certain aspects of perspective appear to be encoded morphosyntactically in lexical or grammatical forms. In Japanese, for example, there is a well-known lexical contrast between verbs of giving (24). Although both verbs lexically describe a giving relation, they do so from a different perspective. The verb *yatta* is said to be appropriate in situations described from the point of view of the subject (Taroo), whereas *kureta* is used to describe the event from the point of view of the dative object (Hanako); see Kuno and Kaburaki (1977) among others for discussion.


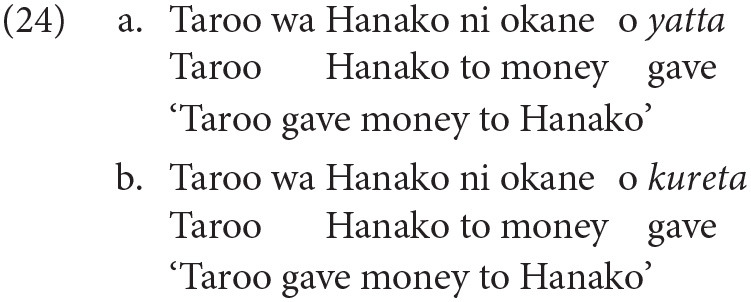


Another example of grammatically encoded perspective comes from German, which employs a dedicated marker of reportive mood, sometimes called *Konjunktiv I* (e.g., Zifonun et al., 1997; Sode, 2014) or the *reportive subjunctive* (Fabricius-Hansen and Sæbø, 2004). It conveys that the speaker is not committed to the content of the clause, and is licensed when subordinate to a verb of saying like *behauptete* (claimed), or for extended narrative discourse, in which a non-speaker viewpoint is reported.


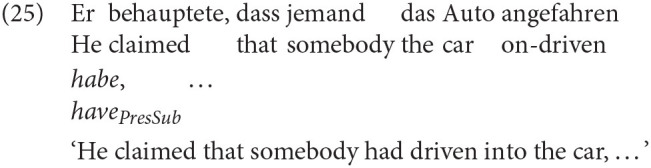


Although English lacks morphological indicators of a particular perspective, certain tense-aspect combinations are sometimes counted among those cues that signal non-speaker perspective (e.g., Smith, 2003). Such combinations will be designated Type B cues. Whereas Caenepeel and Sandström (1992) argue that the past progressive introduces a perspective, I'll assume that tense-aspect configurations do not so much initiate a perspective shift as *maintain* a shifted perspective already active in the previous context. As such, nothing special needs to be said about such cases, as they simply follow from what we already know about how tense and aspect interact with the linguistic context. In this way, Type B cues are elements that are responsible for maintaining a perspective, once shifted. These cases will be referred to as extended shifts of perspective.

A simplified version of a relatively standard three parameter account of tense and aspect is adopted for illustration. Only enough to follow the logic of the experimental design is presented, and many important developments and complications are omitted. Following Reichenbach (1947), Klein (1994), and many others, *tense* encodes the relationship between two temporal intervals: the TU (the time of utterance) and the TTop (a contextually determined topic time). Ignoring the future tense for simplicity, the present tense is used when TU and TTop are simultaneous or overlap (TU = TTop), whereas the past tense is used when TTop precedes TU (TTop < TU). *Aspect* encodes the relationship between TTop and TSit (the time that the event described by the clause occurred). In perfective aspect forms, for example, TTop is said to contain TSit (TSit ⊂ TTop).^4^ The basic ingredients of the system are illustrated with the Past and Present perfect sentences in (26).


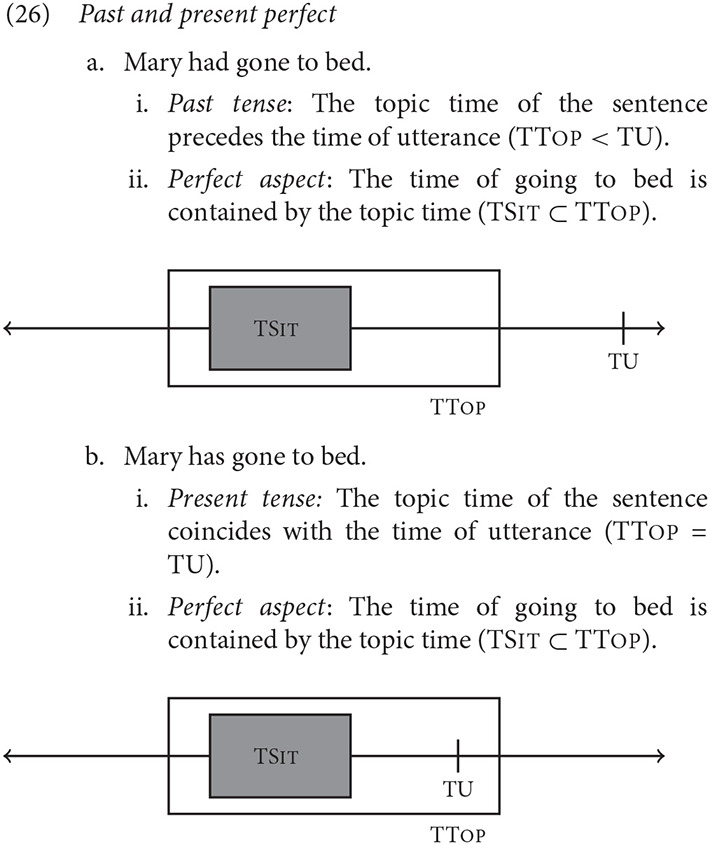


In both cases, the event of going to bed had already been initiated prior to the end of TTop. The only semantic difference is whether TU and TTop overlap. With a few key exceptions relevant to narration, TU is anchored to the time of the present experience. However, the temporal location of TTop depends on contextual information, and can be anchored to events in the narrative sequence. Klein (1994) adopted the view that sentences make reference to a topic situation (Kratzer, 2008), and analogized TTop to the time span associated with it. The TTop can be provided by explicit linguistic context, such as a preceding sentence or a frame or temporal adverbial, but is usually left implicit (Partee, 1973). In the Present perfect, TTop may anchor to TU, though other reference points are possible. However, in the Past, sentences like (26a) seem incomplete without a contextually relevant topic time, e.g., *Sally came home at midnight. John had gone to bed*.

Tense interacts with perspective in intriguing ways. Intuitively, the interpretation of a sentence following a perspective shift depends on the tense and aspect of the continuation. To take the second sentences in (27) as an example, while the Past perfect version (27a) can be interpreted either as speaker or non-speaker oriented, the Present perfect counterpart (27b) is more readily understood as a representing a non-speaker experience.


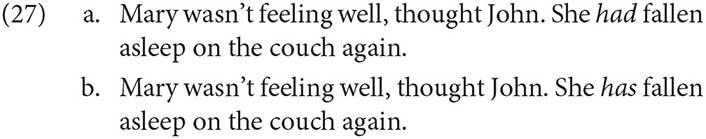


An extended shift in these cases may be achieved by somewhat different means. Shifting in the Past perfect case plausibly represents an instance of classic FID, in which the perspective of the main character is cast as anterior to TU. In the Present perfect case, however, the TU overlaps with TTop, which semantically contains TSit. Provided that the second sentence rhetorically expands on the reported event, the perspective of the character seemingly extends beyond the initial report.

The difference may also be described in terms of how the “contextual now,” a contextually given reference point for the perspectival center, is established. An extended shift in the Past perfect would require the comprehender to infer that the contextual now remains in the present experience of the parenthetical subject. Such a reading is made more accessible when additional Type A indicators of non-speaker perspective are provided (28).


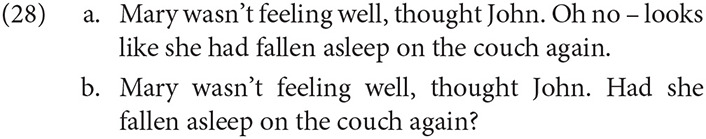


In the Present perfect case, however, a shifted contextual now can be established directly as the TU via the semantics of the Present.

There is a long tradition of positing a shift in TU to capture atypical uses of tense in vivid narrative forms (Reichenbach, 1947; Partee, 1973, 1984; Kamp and Rohrer, 1983; Comrie, 1985; Fleischman, 1990; Hornstein, 1990; Caenepeel, 1995; Michaelis, 1998, among many others). One well-known example among many is an account of the historical present in which TU is back-shifted to the period described in the narrative:


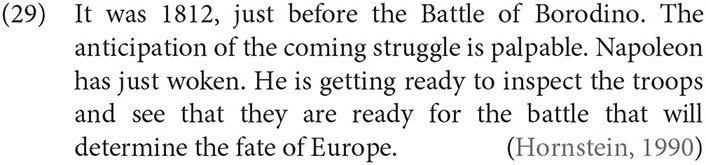


A similar case can be made for future-in-past, in which the contextual now can be understood as situated within an event described in the past (Kaufmann, 2005; Eckardt, 2017).


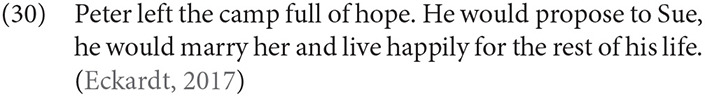


Although I will remain agnostic about the precise mechanisms behind perspective shift in these cases, I hope that the intuitions are clear. What remains now is to provide an account of how the two kinds of cues might conspire to signal and maintain extended perspective shift within the human language processing system.

### 2.2. Processing Perspective

The fact that disparate and complex linguistic information signals perspective raises an important series of questions for language processing research, of which just three are mentioned below. First, how does the processing system represent perspectival information about attitude holders? Second, is this information difficult to access and deploy during sentence comprehension? That is, is calculating and shifting a perspective center costly? Finally, once adopted, is perspective shift costly to maintain over longer stretches of text and discourse?

While these questions will not be addressed in any significant depth here, a basic picture is emerging. Evidence from numerous experimental studies suggests that comprehenders track the knowledge state and (presumed) viewpoint of attitude holders both in live conversation and in text. An early strand of research concentrated on mismatches between *common ground* information, i.e., information which other discourse agents are expected to know, and *privileged ground information*, i.e., information which is private to some individual or subgroup of the community, but not to all members of a discourse. Studies from Boaz Keysar and colleagues argue against an information processing system in which common ground information predominates over privileged ground. According to the monitoring and adjustment (Horton and Keysar, 1996) and the perspective adjustment (Keysar et al., 2000) models, common ground information does not have an impact until later stages of planning and comprehension. That is, language users plan and process utterances *egocentrically* first, only using information from the common ground in later stages to filter out unwanted ambiguities. However, Hanna et al. (2003) found evidence that participants are able to access common ground information early and circumscribe referential domains in a listening task, suggesting a *simultaneous access* view of common ground processing (see also Heller et al., 2008, 2010, and other since).

Further, readers and listeners appear to anticipate characters' likely emotional states while reading. Accordingly, they are surprised when a story does not conform to those expectations (Gernsbacher et al., 1992, 1998). In addition, language users consult a variety of potentially non-linguistic cues from the environment, and appear to generate expectations during interpretation (see Van Berkum (2010) for review). These cues include gender and social class (Van Berkum et al., 2008), political leanings (Van Berkum et al., 2009), and so on. Such information accumulates moment by moment within a representation of context (e.g., Clark, 1996). Thus, it seems clear that, at a minimum, readers and listeners monitor and use alternative perspectives during language comprehension.

How does the language processing system select the appropriate perspective? The discourse-economy approach taken here adopts a presumptive view of perspective processing in which calculating perspective follows a path of least resistance. Instead of continually generating hypotheses about whose perspective the speaker intended to discuss, it simply defaults to the presumption that the speaker is committed to the attitude expressed.


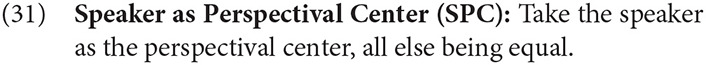


The formulation of the principle highlights the presumptive nature of the proposal. Assuming from the maxim of Quality (Grice, 1975) that cooperative speakers are expected to believe what they assert, it follows that they should expect to be publicly committed to the content of their assertions (see also Levinson, 1983; Atlas, 2005), including presuppositions that project (e.g., Tonhauser et al., 2013). Following a similar logic as Harris and Potts (2009), speakers conveying non-speaker sentiment run the risk of being understood as committing to the perspective, unless they takes pains to signal otherwise. The strategies for signaling non-speaker orientation may vary depending on the genre, with specialized conventions for narrative that differ from other, perhaps more marked, forms of communication.

Although non-speaker uses of an epithet or a socially charged expressive to the incorrect individual carries with it a certain risk, the consequences of misattribution of perspectival terms are sure to vary according to the expression and the context of utterance. For example, the social ramifications of attributing a slur or epithet to the wrong attitude holder are generally greater than misinterpreting a predicate of personal taste. However, cases in which something important hinges on the contextual understanding of *beautiful* or a directional term like *left* can be constructed: for example, emergency instructions to a pilot from an air traffic controller. While these differences raise potentially interesting possibilities regarding whether speakers adjust their strategies according to the consequences of misattribution, the principle behind such cases remains the same: using a perspectival term to express a non-speaker orientation runs the risk of misattribution, leading to a violable preference for speaker-oriented uses.

A strictly speaker-centric view predicts resistance to perspective shifting generally, but has little say about the maintenance of perspetive, however. On the one hand, the processor might be eager to return to the speaker's perspective whenever possible. This view would be broadly compatible with accounts of perspective shifting that require a specialized mechanism for every instance of non-speaker perspective, e.g., a propositional operator that shifts parameters of the context. On the other hand, the processor might follow a path of least resistance when it comes to point of view. In this case, while shifting to a non-speaker perspective might be effortful and require a good deal of evidence, a shifted perspective might not require additional resources to maintain, provided the resulting text is coherent. This view would be broadly compatible with accounts that posit text level operations (e.g., Smith, 2003) or an extra-linguistic representation of perspective (e.g., Harris, 2012). In favor of the second position, I posit another discourse-economy principle favoring processing inertia:


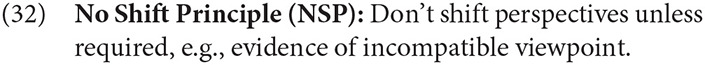


The NSP is predicted to interact with tense through the calculation of the contextual now. Forms like Present tense situate the TU within the contextual now. As long as the content within a sequence of clauses is consistent, the processor should avoid changing the contextual now as a reference point, thus ensuring that the perspectival center remains continuous.

In general, SPC and NSP discourage perspective shift, favoring speaker orientation by default. Once shifted, however, NSP favors to maintain the current perspective, as long the situation described is compatible with the ascribed viewpoint, resulting in an extended shift interpretation. Both principles can be understood as economical in the sense that they advocate against making potentially unnecessary updates to the discourse representation without evidence, but neither one prevents such updates from occurring, even rapidly.

Three experiments were designed to test how the availability of extended perspective shift might vary as a function of report type and tense. Attitudes were introduced with either Narrative parenthetical reports or Standard reports, and the continuation sentence following the report was presented in either Past or Present perfect.


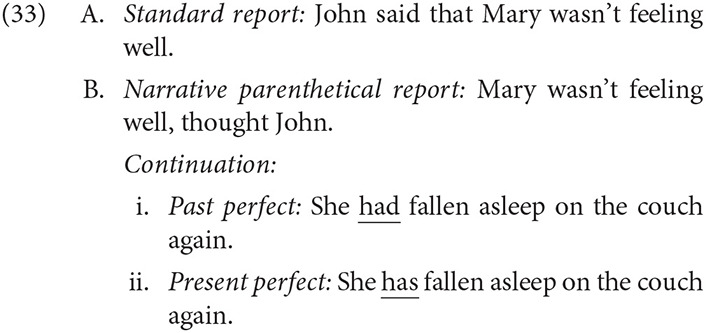


The central prediction was that extended shift interpretations of a Present perfect continuation would be more accessible when following a Narrative parenthetical (33B.ii). Further, if changing perspective exacts a processing cost, even with sufficient evidence for a shift, these continuations should be faster to process and interpret, as they allow the processor to maintain a single, shifted perspective.

## 3. Experiment 1: Two-Person Production-Listening Study

This experiment was designed to test the effect of tense on the interpretation of a sentence following different types of report. If the SPC is correct, then Narrative parenthetical reports should elicit more extended shift interpretations in perspective than Standard counterparts. According to the NSP, the availability of extended shifts should be increased by Present tense in the continuation, by allowing the contextual now to be uninterrupted, which results, by hypothesis, in a non-speaker perspective.

### 3.1. Participants

Fifteen pairs of undergraduate students at the University of Massachusetts Amherst participated in this study, for a total of 30 subjects. Participants were recruited from the Psychology department's subject pool and were offered class credit for their time. Upon entering the testing room, a Producer and a Listener was selected from the pair at random. Producers and Listeners were seated at separate computers, facing away from each other, so that non-verbal communication, such as facial cues and gestures thought to facilitate perspective shift (Harris and Potts, 2011), would not be a factor in the task.

### 3.2. Materials and Methods

The experiment consisted of 21 triplets in three conditions: *Standard report - Past tense* (34a), *Narrative parenthetical report - Past tense* (34b), and *Narrative parenthetical report - Present tense* (34c).^5^ Labels are provided below for convenience and were not shown to participants. Each item consisted of a Report and a Continuation, and were followed by an interpretation question probing the viewpoint of the second sentence (35). Materials are provided in Appendix A.


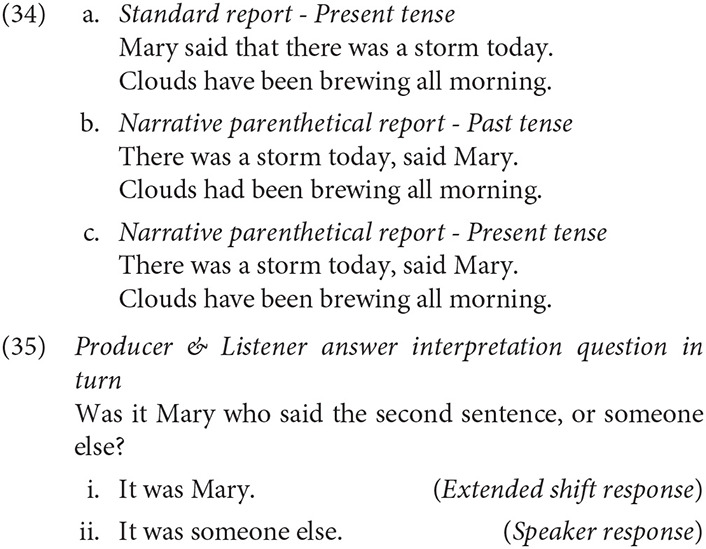


Items were interspersed with 33 items from two unrelated experiments. All items in the study probed perspective or commitment in some form or another. Experimental materials were presented in randomized counterbalanced order, so that pairs saw or heard only one item from each triplet.

Participants were instructed verbally before the experiment began, as well as in written instructions printed on the computer screen. The procedure for Producers and Listeners was somewhat different. Producers were first presented with the target sentence pairs. After reading the sentences, they were asked an interpretation question. After answering the question, they pressed a key to reveal the target sentence again, at which point the Producer read the target out loud to the Listener. Producers were instructed to “perform” the sentences, rather than to simply read them. All productions were recorded by a headset microphone connected to the computer.^6^ After the Producer finished speaking, the Listener was presented with an interpretation question, much like the one that the Producer answered. Participants were allowed to break whenever desired, and typically finished the experiment in less than an hour.

### 3.3. Results

The response data was analyzed as a logistic linear mixed effect regression models with Condition as the fixed effect predictor. After models with maximal random effect models failed to converge (Barr et al., 2013), a model with by-subject and by-item random intercepts was computed. Predictors were treatment coded, so that each level was compared against Standard report - Present tense as the reference level.^7^ The difference between Standard report—Present tense (*M* = 51%, *SE* = 5) and Narrative parenthetical report—Past tense (*M* = 54%, *SE* = 5) conditions was not significant, *z* < 1. However, the Narrative parenthetical report—Present tense condition (*M* = 73%, *SE* = 5) prompted more extended shift responses than the Standard report—Present tense condition, *z* = 5.21, *p* < 0.001.

An additional, exploratory model was constructed in which the role of the participant was added as a predictor. The Narrative parenthetical report—Past tense condition was again the most likely to be associated with an extended shift interpretation, *z* = 4.71, *p* < 0.001. There was a numerical trend toward an interaction with this condition and Participant role, in which Listeners were marginally more likely to give an extended shift response than Producers were, *z* = 1.68, *p* = 0.09.

Participants agreed on their interpretations in 64% of trials in this study and in 62% of trials in the experiment overall. While the extent to which participants agreed on the interpretation of the experimental item did not differ significantly over conditions, there was a numerical trend in which the Narrative parenthetical report -Present tense condition (*M* = 70%, *SE* = 5) elicited greater agreement, *z* = 1.64, *p* = 0.10, than Narrative parenthetical-Past (*M* = 59%, *SE* = 5). Neither of the Narrative parenthetical conditions differed from the Standard-Present (*M* = 65%, *SE* = 5) condition in terms of agreement rates. Limiting the data to trials in which Participants agreed—i.e., the 64% percent of trials in which Producer and Listener judged the token the same, we observe a similar effect as before: extended shift responses were more likely in the Narrative parenthetical-Present condition than in the others, *z* = 3.87, *p* < 0.001, and no other effects were observed.

### 3.4. Discussion

The experiment above investigated the effects of Present and Past perfect progressive sentences following different kinds of report contexts. This primary purpose was to test the basic intuition that the Present tense would prompt more extended perspective shifts when following a Narrative parenthetical than when following a Standard report. The reliable increase of extended shift responses in the Narrative parenthetical report - Present tense condition suggests that neither a Narrative report nor Present tense is sufficient to reliably signal an extended shift on its own. In addition, there was a marginal increase in agreement between participants when multiple cues converge, supporting the idea that multiple cues can increase the efficacy of signaling a perspective shift (Harris and Potts, 2009, 2011; Smith, 2009). However, the experiment lacked a Standard report - Past tense condition, preventing a full exploration of how the conditions may have interacted. The next study reports the results of a fully crossed designed in a interpretation judgment study to assess whether the interaction between condition is superadditive—i.e., the effects of each condition are greater than the sum of the effect of each condition individually.

## 4. Experiment 2: Interpretation Studies

This experiment follows up on the previous experiment by adding a fourth condition to make a fully crossed design. The method was simplified to a standard single-participant forced-choice interpretation task. The central prediction was again that extended perspective shifts would be more likely in Present tense sentences following Narrative parenthetical reports, compared to all other conditions.

### 4.1. Participants

Thirty-six self-reported native speakers of English were recruited online from Amazon Mechanical Turk and were compensated with $6 for their participation. Only subjects who had performed at least 50 previous assignments and received a 98% approval rating or above were permitted to participate in the experiment.

### 4.2. Materials and Methods

The design consisted of 16 quartets crossing Report type (Standard report, Parenthetical report), which was always presented in past tense, and Tense (Past, Present) of the Target sentence. Aspect for these sentences varied across conditions, but were always the same within a quartet. See Appendix B for materials.


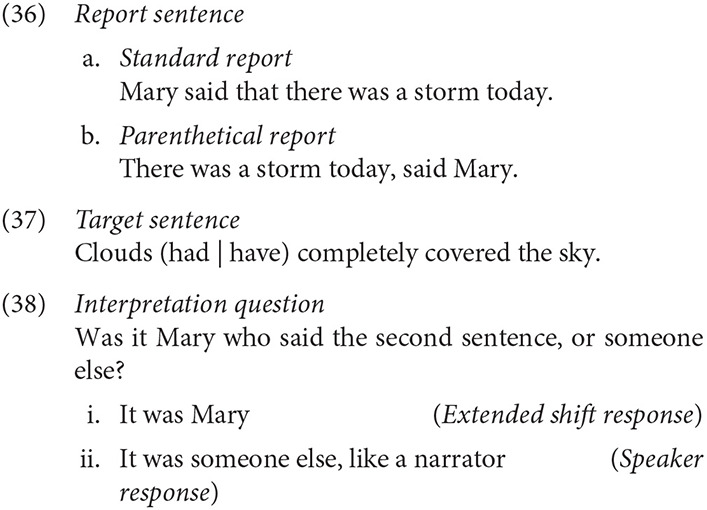


Materials were presented in the second half of a two-part study, and followed a sentence judgment task testing unrelated manipulations. The second part consisted solely of a forced-choice judgment task. Some of the items probed perspectival information. Experimental materials were interspersed with 20 items from unrelated experiments, and presented in counterbalanced and individually randomized order.

### 4.3. Results

**Figure 2:**
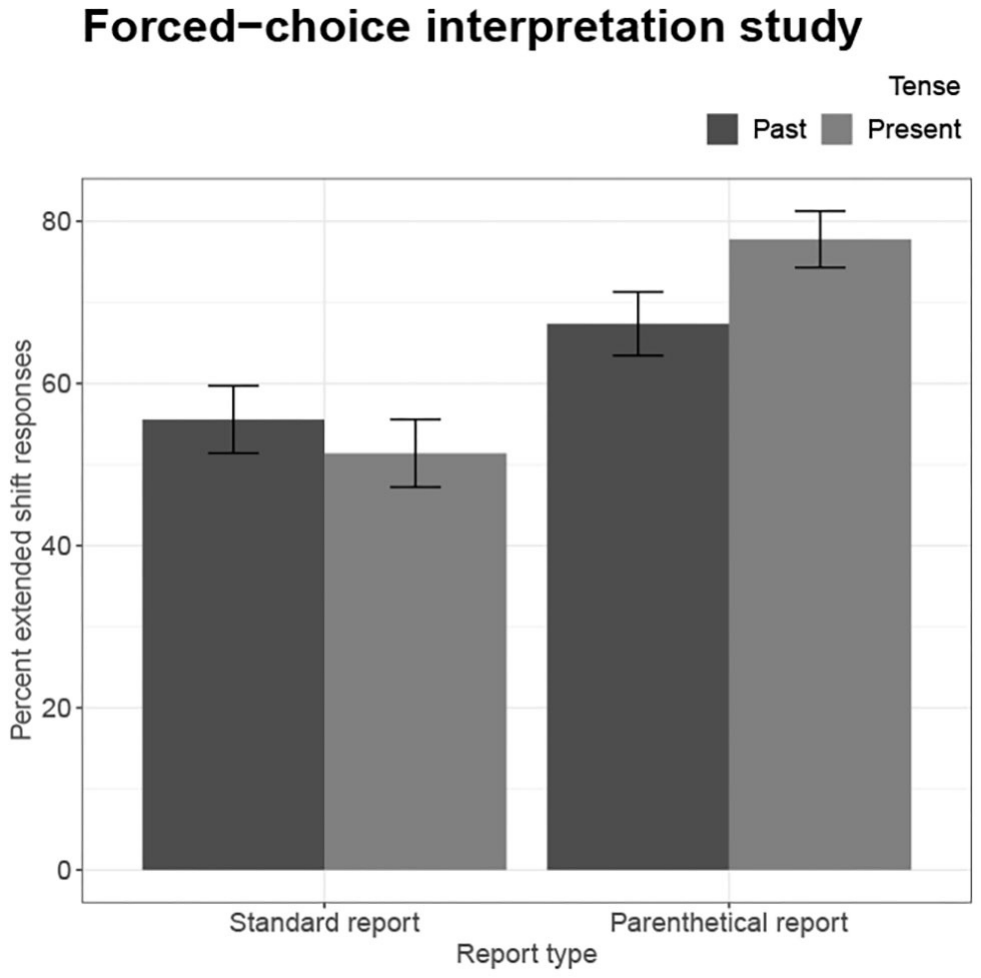
Experiment 2: Forced-choice interpretation study.

The mean percent of extended shift responses and standard errors are provided in Table 1 and depicted in Figure 2. The data were analyzed as linear mixed effect regression models with Report type, Tense, and their interaction as fixed effects and by-subject and by-items random intercepts, after a model with maximal random effects structures did not converge. Predictors were sum-coded so that Standard report and Past tense conditions served as the statistical baseline.

**Table 1:** Experiment 2: mean percent and standard error of extended shift responses for each condition.

	**Tense**	
**Report type**	**Past**	**Present**
Standard	56% (4)	51% (4)
Parenthetical	67% (4)	78% (3)

Parenthetical reports (*M* = 73%, *SE* = 3) elicited more extended shift interpretations than Standard reports (*M* = 53%, *SE* = 3), *z* = 5.62, *p* < 0.001. Although there was no general effect of Tense, Report type and Tense conditions interacted, *z* = 2.40, *p* < 0.05. Present tense targets following Parenthetical reports increased extended shift responses by 11 percentage points, but decreased these responses by 5 percentage points following Standard reports.^8^

### 4.4. Discussion

The results can be summarized by two central findings. First, Narrative parenthetical reports elicited more extended shift responses than Standard reports did. Second, Present tense in a continuation had a greater effect on interpretation when following a Narrative parenthetical report, supporting the idea that the effect of Narrative parentheticals and Tense on perspective is greater than the effect of either condition individually.

In this experiment and the previous one, there were relatively high proportions of extended shifted interpretations across the board. This is perhaps unexpected given the predictions of the SPC, which biases toward a speaker viewpoint. Several explanations are possible. The first is that the speaker default is empirically incorrect. A second possibility is that the proportion of shifted interpretations is partially artificial and due to experimental factors. For example, the questions may have been unclear, introducing additional noise into the responses. Given that a shifted interpretation was selected in approximately half of the trials, participants may have been more prone to guess on these trials. Further, as other items in both of these experiments probed perspective, subjects may have become desensitized to the speaker default, opting for shifted perspective responses more often. This issue was addressed in the third, and final, experiment.

## 5. Experiment 3: Self-Paced Reading Study

This study was designed with several objectives in mind. First, the previous experiments were conducted using offline measures, which do not provide temporal information about how long participants read the passages or spent on the interpretation questions. According to an economy-based account, maintaining a shifted perspective is preferred and shifting perspective, even back to the speaker, exacts a cognitive cost. If correct, this account predicts a processing advantage for Present tense continuations following a Narrative parenthetical report. The central purpose of this experiment was to test this prediction.

Second, in the previous experiments, the reports were presented in Past tense regardless of the report type. Therefore, continuations in Present tense did not match with the tense of the report. It is possible that the mismatching tense was perceived as a marked narrative progression, leading participants to seek a non-standard extended shift interpretation (see Fludernik, 1993, for comments on the relationship between markedness and perspective). Although this explanation of Experiment 2 is unlikely, given that there was no independent effect of tense, it does raise the possibility of an alternate mechanism for extended perspective shift. In the third experiment, the tenses in the report and continuation always matched. The central prediction is again that the interpretation of Present tense continuations would be differentially affected by Report type, resulting in increased extended shift responses for Present tense continuations after a Narrative parenthetical.

Finally, to address potential habituation to non-speaker perspectives, no other items involving perspective shift were included, and interpretation questions were presented after only half the items. If the response rates observed in Experiments 1 and 2 were due, at least in part, to saturation with shifted perspectives, then the overall rate of extended shifts should be reduced.

### 5.1. Participants

Forty-eight subjects were recruited from the Claremont Colleges and compensated with course credit or $10 cash. All subjects self-reported as native speakers of English.

### 5.2. Materials and Methods

Materials consisted of 16 two-sentence discourses (39) modeled after those in Experiment 2. Conditions crossed Report type (Standard report, Parenthetical report) and Tense (Past, Present). The tense of the report and the continuation were matched, e.g., *was* paired with *had* and *is* with *has* or *have*, to avoid any effects of tense mismatch. Interpretation questions like (40) below were presented after half of the items.

Discourses were presented in a self-paced non-cumulative moving window fashion. The first sentence was always presented in its entirety on the first line. The second sentence was presented on region by region on the following line, as demarcated in (39). Regions of interest were identified as those that encode tense and/or aspect information: *Auxiliary* (Region 2), *Perfect participle* (Region 3), and *Verbal participle* (Region 4). Interpretation questions (40) followed half of the items to reduce participant saturation.


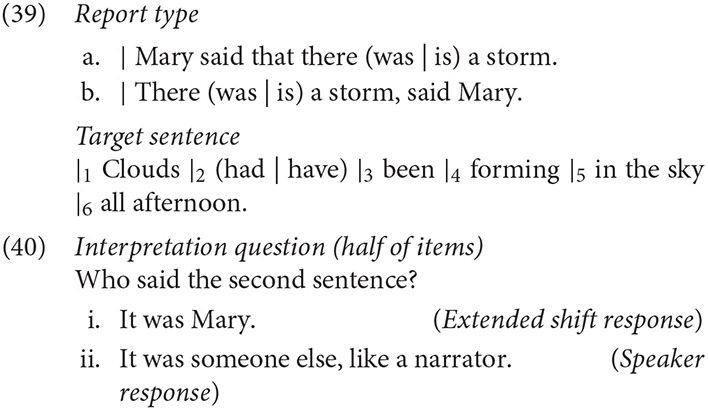


Sentences were counterbalanced, so that each subject saw only one condition from the quartet. Items were presented in individually randomized order and interspersed with 66 items from unrelated experiments, 36 of which were also presented on two lines, and 28 non-experimental filler items, 12 of which were presented on two lines. None of these items were associated with perspective shift. The experiment lasted approximately 40 min on average per participant.

### 5.3. Results

Prior to analysis, outliers were censored according to a procedure known as winsorization (Dixon, 1960; Tukey, 1962). Observations above the bottom and top 5th percentile were transformed to the 5th and 95th percentile, respectively. The results did not differ in kind from those obtained with untransformed data. Adjusted means and standard errors for all regions are provided in Table 2. The second row of the table lists the mean time subjects spent reading the interpretation question and making a decision, as well as the percent of responses indicating an extended perspective shift.

**Table 2:** Experiment 3: self-paced reading means and standard errors in parentheses.

		**Region**						
**Report type**	**Report**	**1: Report**	**2: Subject**	**3: Auxiliary**	**4: Perfect**	**5: Verbal**	**6: Spillover**	**7: Final**
	**Tense**				**Participle**	**Participle**		
Standard	Past	1939 (38)	570 (20)	419 (12)	388 (11)	410 (11)	486 (15)	677 (34)
	Present	1974 (39)	597 (24)	438 (18)	392 (11)	445 (18)	486 (14)	700 (31)
Parenthetical	Past	1774 (35)	584 (19)	411 (9)	400 (14)	443 (15)	491 (15)	714 (34)
	Present	1637 (31)	565 (15)	401 (11)	380 (8)	408 (11)	493 (16)	681 (30)
		**Decision time**	**Extended shift**					
		**on question**	**responses**					
Standard	Past	2,242 (38)	34% (5)					
	Present	2,244 (43)	47% (5)					
Parenthetical	Past	2,024 (43)	44% (5)					
	Present	1,891 (32)	81% (4)					

Linear mixed effect regression models with Report type, Tense, and their interaction as fixed effects were created for regions of interest. Conditions were sum-coded as in Experiment 2. Decision time and responses to the interpretation question were modeled separately. Random effects were specified as by-subject and by-item random intercepts, after models with random slopes failed to converge. When interactions between conditions were significant in reaction time measures, the estimated marginal means of the model for Past and Present tenses were compared within each Report type using the *emmeans* package (Lenth, 2019), for which model estimates and 95% confidence intervals are reported. All significant effects are reported.

#### 5.3.1. Reading Times on Regions of Interest

**Figure 3:**
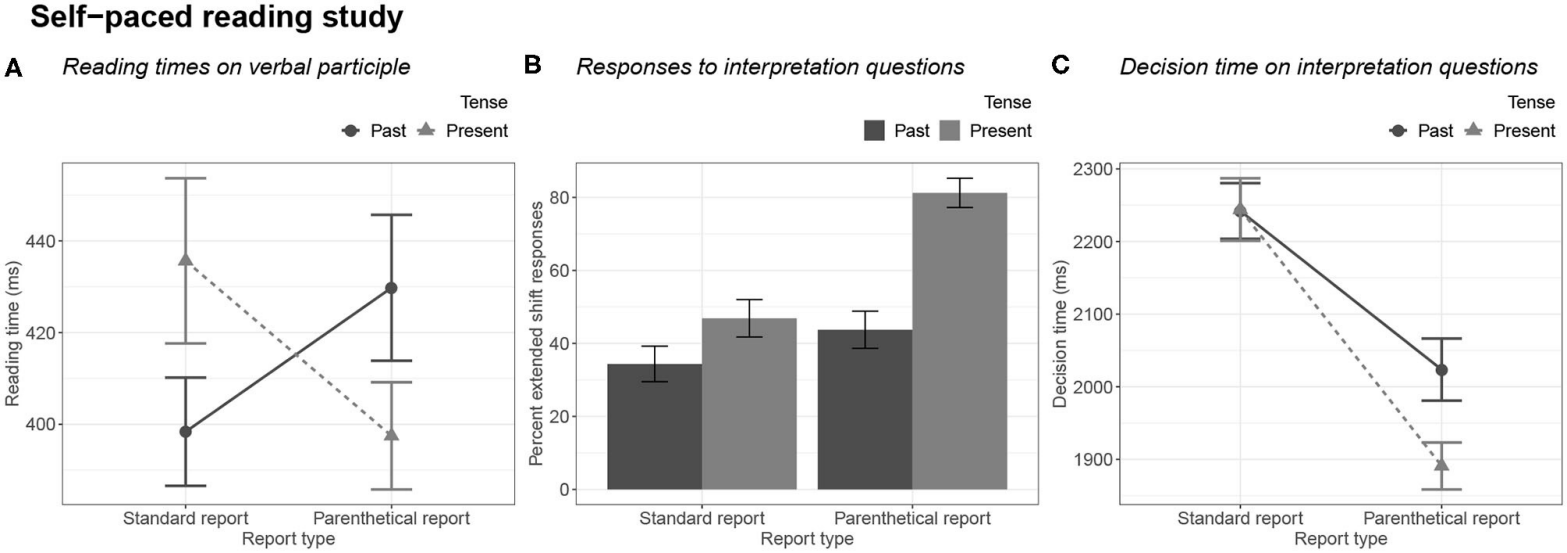
Experiment 3: Self-paced reading study. **(A)** Reading times on verbal participle. **(B)** Reading times on interpretation questions. **(C)** Decision time on interpretation questions.

Statistical models are provided in Table 3. On the auxiliary (*has / have* or *had*) region, there was a general advantage for Parenthetical (*M* = 404, *SE* = 7) over Standard reports (*M* = 425, *SE* = 10), *t* = −2.31, *p* < 0.05. No effects were observed on the perfect participle (*been*) region. On the verbal particle (*V-ing*) region, there was a crossed interaction, *t* = −3.13, *p* < 0.001; see Figure 3A. While Present tense elicited a 38ms penalty in Standard reports (*Marginal means:* β = 34.80, *SE* = 15.90, 95% CI [3.62, 66.06], *p* < 0.05), it elicited a 33ms advantage in Narrative parenthetical reports (*Marginal means:* β = −35.50, *SE* = 15.90, 95% CI [−66.73, −4.28], *p* < 0.05).

**Table 3:** Experiment 3: linear mixed effect regression models for regions of interest.

**Region**	**Parameter**	**Estimate**	**Std. Error**	**t-value**	**p-estimate**
Auxiliary	(Intercept)	414.60	16.14	25.69	<0.001
	Parenthetical	−10.67	4.62	−2.31	<0.05
	Present	0.25	4.62	0.06	0.96
	Parenthetical × Tense	−6.58	4.62	−1.42	0.15
Perfect	(Intercept)	388.47	15.51	25.05	<0.001
participle	Parenthetical	−0.70	3.86	−0.18	0.86
	Present	−3.41	3.86	−0.88	0.38
	Parenthetical × Tense	−5.37	3.86	−1.39	0.17
Verbal	(Intercept)	426.51	18.02	23.67	<0.001
participle	Parenthetical	−0.84	5.62	−0.15	0.88
	Present	0.17	5.62	0.03	0.98
	Parenthetical × Tense	−17.59	5.62	−3.13	<0.001

#### 5.3.2. Interpretation Questions

Statistical models are provided in Table 4. Analysis of the interpretation questions revealed that Parenthetical reports were associated with increased extended shift responses, *z* = 5.00, *p* < 0.001, as was Present tense, *z* = 5.54, *p* < 0.001. As illustrated in Figure 3B, Present tense resulted in a greater increase in extended shift responses in the Narrative parenthetical report condition (diff = 37%) than in the Standard report condition (diff = 13%), *z* = 3.03, *p* < 0.001.

**Table 4:** Experiment 3: linear mixed effect regression models for responses to and time spent on interpretation questions.

**Measure**	**Parameter**	**Estimate**	**Std**.	***t*/*z*−value**	**p-estimate**
			**Error**		
Responses	(Intercept)	0.15	0.30	0.49	0.63
	Parenthetical	0.70	0.14	5.00	<0.001
	Present	0.78	0.14	5.54	<0.001
	Parenthetical × Present	0.41	0.13	3.03	<0.001
Decision time	(Intercept)	2100.15	33.22	63.22	<0.001
	Parenthetical	−142.89	17.81	−8.02	<0.001
	Present	−32.66	17.81	−1.83	0.07
	Parenthetical × Present	−33.70	17.81	−1.89	0.06

Decision times on interpretation questions showed that subjects were faster to read and respond to Narrative parenthetical report conditions, *t* = −8.02, *p* < 0.001, and were marginally faster in Present tense conditions, *t* = −1.83, *p* = 0.07. These conditions marginally interacted, in that there was a greater effect of Tense on Parenthetical reports than on Standard reports, *t* = −1.89, *p* = .06. Planned comparisons revealed a 133ms advantage for Present Narrative parenthetical reports (*Marginal means:* β = 132.72, *SE* = 50.4, 95% CI [33.6, 232], *p* < 0.01), but only a 2ms difference in Standard reports; see Figure 3C.^9^

### 5.4. Discussion

The responses to interpretation questions patterned very closely with the central results from the previous two experiments. Present tense continuations following Narrative parenthetical reports were associated with increased extended shift responses at a greater rate than Past tense continuations were. As the tense in the report and the continuation matched, the interaction cannot be attributed to tense mismatch leading to increased extended shift interpretations as a result of a more marked sequence. In addition, the percentage of extended shifts in conditions other than the Narrative parenthetical - Present tense condition was reduced, suggesting that the high rates observed in Experiments 1 and 2 may be partially attributed to experimental artifacts.

Decision times on interpretation questions also revealed an interaction between conditions, showing a greater advantage for Present tense continuations following a Narrative parenthetical report compared to a Standard report. Both of these results are highly compatible with the NSP, in that a non-speaker perspective after a perspective shift was predicted to be the preferred, and most economical, interpretation.

This approach made a similar prediction for the online, incremental processing of perspective. The expected interaction was observed, as participants read Present tense continuations faster after a Narrative parenthetical. However, the effect appeared on the verbal particle, two words after the morpheme marked for tense. While a delayed effect is very common in self-paced reading studies (Mitchell, 2004), it should be noted that the two previous regions trended in the same direction. The interaction is again compatible with the predictions of the discourse-economy account, in which a maintaining a non-speaker perspective is preferred after a perspective shift.

In addition, the interaction revealed an unexpected cost for Present tense continuations following Standard reports. To speculate briefly, it is possible that this effect reflects a pragmatic clash regarding the speaker's presumed epistemic warrant and discourse coherence. Let's assume that the Present tense, when anchored to a speaker perspective, indicates that the speaker has enough reliable evidence about the ongoing situation to license a statement about it. For example, the speaker would be taken as the evidential source for the statement *Clouds have been forming all morning*. However, one use of a Standard report is to mark the attitude holder as the source for report (e.g., Simons, 2007), resulting in a mild pragmatic clash. A similar clash would not arise in Past tense cases as there is no inference that the speaker has acquaintance with the events described, at least beyond what has been reported to her. Although this explanation remains speculative, it presents a possible avenue for exploring the role of discourse coherence relations between sentences in future studies.

## 6. General Discussion

In this paper, we have concentrated on the phenomenon of *perspective shift*, in which a sentence (or sub-sentential constituent) may be presented as reflecting the perspective of an attitude holder other than the speaker. The topic has received increased attention in the theoretical linguistics literature, where several different subtypes of perspective shifting, and analyses thereof, have been proposed. Interest has also increased steadily among researchers in psychology and psycholinguistics, as a growing body of research suggests that perspectives are tracked and utilized during language comprehension.

I've sketched the outlines of an economy-based discourse model encapsulated by two basic principles. First, I proposed a general speaker default (Speaker as Perspectival Center; SPC), in which shifting away from a speaker's perspective requires ample evidence (following Smith, 2003; Potts's, 2005; Harris and Potts, 2009, among others). This principle serves to constrain perspective shift to cases where the speaker has provided the audience with sufficient cues to retrieve the intended message. Second, the No Shift Principle (NSP) proposed that perspectival continuity is preferred whenever possible. Together, the two principles predict that although shifting to a non-speaker perspective tends to be avoided, it is preferred after shifting has occurred. The principles converge on the idea that the processor doesn't continually assess perspective, but instead shifts only as required. Theoretically, this reluctance to shift results in making processing perspective more efficient.

The central predictions of the model were supported in three experiments. Following Reinhart (1975, 1983) and others, Narrative parenthetical reports were assumed to signal a non-speaker source for the reported content. Sentences following the report were interpreted from a non-speaker perspective at a greater rate when presented in Present than in Past tense. The result was interpreted as a preference to continue the non-speaker perspective from the report in an extended shift interpretation. An online advantage for these sequences was also observed in a self-paced reading study.

Multiple cues indicating perspective and perspective shift have been discussed in the literature (e.g., Banfield, 1982; Wiebe, 1994; Smith, 2003). I proposed two classes of cues characterized by whether they (i) are associated with a perspective as an evaluator through quasi-conventional means (Type A), or (ii) grammatically determine how events are represented (Type B). I suggested that these two types are further distinguished by their primary role of establishing or maintaining a perspective shift, respectively. Following Smith (2003) and Harris and Potts (2009, 2011), the experimental results indicate that cues to perspective might work together to magnify the effect of perspective tracking. This observation leads to a natural follow up to determine whether additional perspectival devices facilitate perspective shift even further, and whether Type A cues may also serve to maintain an extended perspective shift, as discussed in connection with (28) in section 2.1.2.

The experiments presented here addressed whether the way an attitude is presented interacts with the tense of the following sentence in maintaining a shifted perspective. As the results were replicated across several different experimental paradigms with different subjects and filler sentences, the findings are likely to be fairly robust. However, the scope of the manipulations remained relatively constrained, and it remains to be seen whether the processing principles extend to other tenses, constructions, or perspectival cues, and, in particular, whether such cues also interact in extended perspective shift. As speakers may be wary of perspectival information “leaking” into their own public commitments (Lasersohn, 2007; Potts, 2007), they may tend to signal a non-speaker perspective with multiple cues to increase recoverability, particularly when the content is, in some way, important or controversial. Although how perspectival cues might interact remains an open question, existing evidence suggests that comprehenders are sensitive to multiple, interacting cues.

This issue was addressed in part by Harris and Potts (2009), who investigated whether appositive relative clauses, underlined in (41a–b), could shift to a non-speaker perspective when syntactically embedded under a verb of saying (Schlenker, 2007, 2009). Although embedding did increase the likelihood of perspective shift, they found that items containing an evaluative predicate like *paranoid* in the context were not only associated with more perspective shifted interpretations, but also elicited higher rates of perspective shift when embedded, compared to items without evaluative terms.


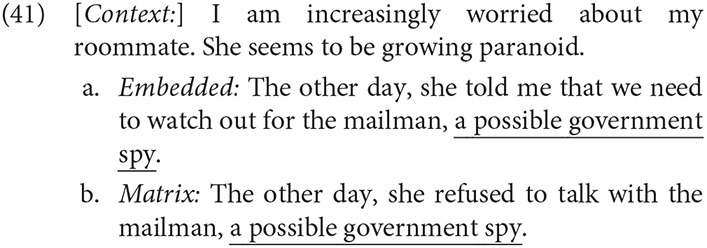


Harris and Potts (2009) concluded that multiple factors contribute to non-speaker interpretations of subjective content, and that the combination of these factors seems to strengthen perspective shift. They proposed that non-speaker interpretations were marked as non-conventional and required additional evidence to shift perspective. Harris (2012, p. 13) further argues that the speaker default, and its violations, can be understood in terms of markedness, along the lines of Horn (1984):

The distribution of speaker-orientation thus appears to follow a distribution of markedness. Speakers use expressions that are conventionally more marked when attempting to convey unfamiliar or otherwise less accessible meanings, at the cost of brevity dictated by the maxim of Manner. It is fairly natural to treat this distribution in terms of a default which enjoins the hearer to interpret a clause as speaker-oriented, unless there is specific evidence to the contrary [(31)].

Crucial to a successful perspective shift is the identification of a prominent discourse agent who can be associated with the shifted perspective (e.g., Hinterwimmer, 2019). Salem et al. (2018) conducted two online experiments investigating whether discourse particles and rhetorical questions increased the salience of a protagonist attitude holder in German. Increased salience was hypothesized to reduce time readers spent on an anaphor referring to the protagonist in passages of FID text. However, these markers did not influence the speed at which readers processed the anaphor online, and failed to reliably affect speed or accuracy on comprehension questions following the experimental passage. Such results would seem to contrast with those reported in Experiment 3.

It is possible that the accessibility of perspective shift is highly dependent on experimental design factors. The question of cue interaction was the subject of an additional exploratory pilot study (*N* = 36). Several different kinds of subjective constructions were presented to subjects to judge whose perspective was being portrayed with instructions similar to Experiment 2. One set of sentences (13 items) were modeled after the past tense conditions of the experiments above, and crossed report type with the presence of an interjection, such as *my goodness*.


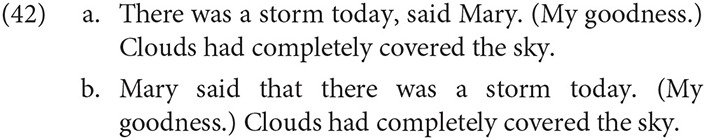


Without an interjection, subjects provided extended shift responses after Narrative parenthetical and Standard report types at equal rates (52%). Interjections increased extended shift responses more for Narrative parenthetical (81%) than Standard (66%) reports, β = 0.22, *SE* = 0.11, *z* = 2.03, *p* < 0.05.

Also included in the pilot were passages with experiencer predicates and subjective terms (16 items). Statements were compared with questions in past and present tense:


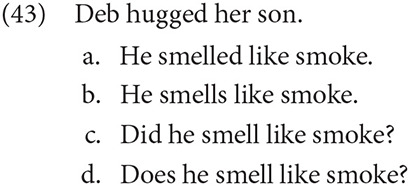


Present tense statements (43a) increased the rate of extended shifts (80%) compared to past tense statements (43a; 58%), compatible with the experiments above. However, tense had no effect on passages containing questions, which appeared to perform at ceiling (90% for both tenses).

While a more complete picture is still emerging, these preliminary results support the claim that calculating perspective relies on multiple, interacting factors. Some cues may exert varying degrees of influence; questions, for example, appear to be strong indicators of a non-speaker orientation, obscuring the effects of tense. This finding is highly compatible with a taxonomical distinction between cues that indicate a shift in perspective and those that maintain a perspective once shifted.

As noted above, perspective shifting has received many treatments. Multiple approaches treat perspective shift in terms of a silent operator which may adjust context-sensitive values in linguistic expressions or by altering parameters within the context. Approaches further differ according to the level of representation that the operator takes scope over. On sentence-based accounts, a context-shifting operator is posited at the sentence level, and presumably must be posited for each shifted sentence. On discourse-based accounts, a single perspective-shifting operator may scope over multiple, connected sentences in a fashion reminiscent to accounts of modal subordination (Roberts, 1989), as in Smith (2003) and Fabricius-Hansen and Sæbø (2004).

At first blush, the discourse-based account would appear to be more compatible with the claim that perspective shifting is avoided if the contextual values are consistent with the shifted viewpoint. In contrast, sentence-based accounts would not necessarily predict that extended perspective shifts would be as accessible as they are, as the processor would need to posit another context-shifting operator at each sentence. However, both approaches were designed to formalize how context-sensitive expressions are resolved within a particular representational framework, and not to make predictions about language processing behavior. To bridge the gap, they would each need to provide a principled account of the conditions under which context-shifting operators are licensed in order to make testable predictions.

Licensing conditions might be captured in various ways: for example, as an algorithm that determines whether a non-speaker perspective is available. Wiebe (1991, 1994) proposed an explicit procedure to determine the perspective of an utterance from features indicating subjectivity in a sentence (e.g., the forms listed in (12)) and properties of the current context, such as the most recent perspectival center. Abrusán (to appear) has recently offered a rich account that integrates this function with Segmented Discourse Representation Theory (Asher et al., 2003), a theory that models the mental states as a discourse context anchored to an attitude holder and the object of belief. This framework allows us to incorporate coherence relations between sentences, as well as the information structural status of referents in text, into an explicit procedure for identifying the perspectival center.

Another approach was detailed by Harris (2012), who proposed that a conversational scoreboard maintains information about discourse agents (Lewis, 1979), including the current perspectival center. The processor was assumed to passively construct a model of situations mentioned and implied by a text and the attitudes of central characters in text (e.g., Garrod and Sanford, 1985, 1995; Sanford and Garrod, 1998). The commitments, attitudes, and likely beliefs of discourse agents are collected into a representation called an “agent profile.” He proposed that values in the scoreboard are updated by a general abductive inferencing procedure that provides the most reasonable interpretation given what is known about discourse agents via their profiles and presentational cues provided in the text. Assuming that abductive inferencing is computationally costly and prone to error, discourse economy provides a shortcut through this expensive inferencing process.

In summary, the three experiments reported here support the idea that Type A cues, like Narrative parenthetical reports, collude with Type B cues, like Tense, to produce an extended perspective shift. And while shifting a perspective away from the speaker may be costly to achieve, maintaining a non-speaker perspective itself may, at times, be the most economical option. In general, the results suggest that the perspectival center is not calculated at each moment in interpretation. Instead, the processor defaults to a speaker perspective unless it encounters evidence promoting a likely shift in interpretation, but may settle on a single perspective for longer stretches of discourse. Although the question of precisely how to model perspective remains open, the studies above support a model of perspective that is subject to general economy considerations, avoiding re-evaluating the perspectival center whenever possible.

## Data Availability Statement

The raw data supporting the conclusions of this article will be made available by the authors, without undue reservation.

## Ethics Statement

The studies involving human participants were reviewed and approved by the Internal Review Boards at the University of Massachusetts Amherst and Pomona College. The patients/participants provided their written informed consent to participate in this study.

## Author Contributions

The author confirms being the sole contributor of this work and has approved it for publication.

## Conflict of Interest

The author declares that the research was conducted in the absence of any commercial or financial relationships that could be construed as a potential conflict of interest.

## Footnotes

^1^For present purposes, I take a commitment to represent “a kind of *persona* of beliefs” (Hamblin, 1970). An individual *X*'s commitment is inferred from the content of a particular expression *E*, and licenses the inference that *X* is committed to the presuppositions associated with *E* (Peters, 2016). Commitments are distinct from *genuine beliefs*, as they may be provisional, disingenuous, or adopted the purposes of the discourse (Hamblin, 1970, 1971). Commitments may also be shared or dependent on another source (e.g., Gunlogson, 2008; Harris, 2014; Malamud and Stephenson, 2014).

^2^The use of the term “individual” may not be entirely accurate, in that predicates of personal taste have been argued to display elements of normativity (Buetow, 2014) or genericity (Moltmann, 2010; Pearson, 2013).

^3^Moore (2011) details how similar devices marked direct speech in early English before the use of quotation marks was standardized. The convergence of these forms is unlikely to be accidental (Fleischman, 1990).

^4^The perfect is extremely complex and has several distinct uses; see Comrie (1976), Klein (1994), and Portner (2011) for discussion. Consideration of these uses is beyond the scope of the present project.

^5^For simplicity, I have ignored complications that arise from the possibility of a “simultaneous” vs. “back-shifted” reading of the past embedded under a past tense verb (e.g., Ogihara and Sharvit, 2012). The sentences were designed to be biased toward a simultaneous reading, in which the event of the subordinate clause (*there was a story toda*y) overlap with the time of the report. The availability of multiple temporal interpretations of the embedded clause could be explored in future studies.

^6^Audio recordings were not analyzed statistically, but were reviewed impressionistically for cues of quotation, e.g., those discussed in Jansen et al. (2001) and Oliveira and Cunha (2004). Although no such cues were observed, a more systemic analysis is required to properly address the issue.

^7^As observed by a reviewer, random intercept linear mixed effects regression models are anti-conservative, and increase the chance of Type I error. Accordingly, Barr et al. (2013) recommend fitting “maximal” random effects structures, with by-subject and by-item random slopes and intercepts whenever possible. However, Matuschek et al. (2017) note that maximal models run the risk of inflating Type II error. To address these issues, Bayesian maximal mixed effect models were computed with brms (Bürkner, 2017), specified with 10,000 iterations and 1,000 warm up cycles. All effects in each of the three experiments exhibited the same pattern as those reported here with a standard 89% credible interval (McElreath, 2020) with the exception of the reaction times on questions in Experiment 3, for which divergent transitions could not be resolved. A simpler random intercepts Bayesian model showed the same pattern as the linear mixed effects regression model. Since the effects were replicated multiple times in different paradigms with different subjects, the effects are likely to be highly robust.

^8^The continuation sentence was mistakenly presented in simple Past and Present, rather than in the Perfect, for three items (6, 7, and 10 in Appendix B). Removing these items did not change the patterns reported above. There was still a main effect of Report type and an interaction between Report type and Tense. The means were nearly identical to those obtained from the entire dataset.

^9^Note that the analysis suffered a considerable loss of power, as there were only 8 interpretation questions per subject. The lack of full significance in a case where the difference between conditions is so large may be the result of an underpowered analysis.
