# Supplementary material for: Extended Perspective Shift and Discourse Economy in Language Processing
Source: Front Psychol. 2021 Mar 31;12:613357. doi: 10.3389/fpsyg.2021.613357 (PMC8044538; doi:10.3389/fpsyg.2021.613357)
Supplement: Supplementary file 1 [file Data_Sheet_1.pdf]

Supplementary Materials: *Extended perspective shift and discourse economy in language processing*.
Jesse A. Harris

1 Materials from Experiment 1.

Materials from Experiment 1. Only the Narrative parenthetical report is provided, as the Standard report condition can be derived from it.

1. There might be a storm today, said Mary.
   Clouds (has | had) been brewing all morning.
2. Snow would come soon, thought Jim.
   It (has | had) been getting colder by the minute.
3. The town must get rain soon, said the villager.
   It (has | had) been dry for months on end.
4. My career is over, cried the politician.
   The media (has | had) been reporting a new scandal every day.
5. The company is in great peril, gasped the CEO.
   The shareholders (have | had) been discussing a hostile takeover.
6. The offense needs to work harder, shouted the coach.
   The team (hasn't | hadn't) won a game all season.
7. It's going to explode, cried the chemist.
   The chemical compound (has | had) rapidly been growing unstable.
8. Something appears to be bothering the animals, said the zookeeper.
   The monkeys (have | had) been shaking the cage all afternoon.
9. The city is under siege, exclaimed the judge.
   Violent crime (have | had) recently been on the rise.
10. A mutiny might break out at any moment, growled the first mate.
    The ship's crew (haven't | hadn't) eaten a good meal in weeks.
11. It's cold in the workshop, admitted the carpenter.
    The heat (has | had) been broken for several weeks.
12. The books are covered in dust, exclaimed the librarian.
    Nobody (has | had) checked them out in decades.
13. Conditions in the field hospital are atrocious, sighed the doctor.
    Rebels (have | had) been stealing the medicine from the supply room.
14. Everyone has been so kind, added the bride.
    The wedding guests (have | had) been giving toasts all night.
15. The church is very blessed, acknowledged the priest.
    The congregation (has | had) been regularly donating to the parish for years.
16. The new play is a great success, bragged the actor.
    Opening night (has | had) been sold out months in advance.
17. The union is ready to go on strike, whispered the assembly worker.
    The factory owners (have | had) been mistreating its workers for a long time.

18. The swamp must have once supported a vibrant ecosystem, concluded the biologist.
    New fossils (have | had) been uncovered every few days.

19. Times are tough for small farms, grumbled the farmer.
    Bigger farms (have | had) been driving out competition everywhere.

20. The National Guard will work with the local police, announced the governor.
    Riots (have | had) been breaking out all over the city.

21. The audience is getting impatient, cautioned the band’s manager.
    The crowd (has | had) been waiting for the show to start for over an hour.

2 Materials from Experiment 2

Experimental materials from Experiment 2. The Standard report condition is omitted.

1. There was a storm today, said Mary.
   Clouds (have | had) completely covered the sky.

2. The snow had come early this year, said Jim.
   It (has | had) been coming down all week.

3. The town was in trouble, said the villager.
   Bandits (have | had) been threatening the townsfolk.

4. The reports were inaccurate, said the politician.
   Newspapers (have | had) been looking for a new a scandal.

5. The company was in great peril, said the CEO.
   The shareholders (have | had) been discussing a hostile takeover.

6. The offense needed to work harder, said the coach.
   The championship (is | was) on the line.

7. The market is up today, said the broker.
   The economy (is | was) finally back on its feet.

8. The animals were frightened, thought the zookeeper.
   The monkeys (have | had) been shaking the cage all afternoon.

9. The city was under siege, thought the judge.
   Violent crime had (has | had) risen to new heights.

10. The crew was acting suspicious, thought the first mate.
    They were clearly unhappy with the captain.

11. It was cold in the workshop, thought the carpenter.
    The heat (has | had) been broken for several weeks.

12. The books were covered in dust, thought the librarian.
    Nobody (has | had) checked them out in decades.

13. Conditions in the field hospital were atrocious, thought the doctor.
    There just (aren’t | weren’t) enough doctors to treat all the patients.

14. The wedding guests were very kind, thought the bride.
    They (has | had) been giving toasts all night.

15. The church was very blessed, thought the priest.
    The steeple (has | had) been entirely rebuilt.

16. The new play is a great success, thought the actor.
    Opening night (has | had) been sold out months in advance.

3 Materials from Experiment 3

Experimental materials from Experiment 3, showing only the Narrative Parenthetical report, as above. Interpretation questions were presented after sentences 1–8.

1. There (is | was) a storm today, said Mary. | Mary said that there (is | was) a storm today.
   Clouds (has | had) been forming in the sky all afternoon.

2. The snow (has | had) come early this year, said Jim.
   Flurries (have | had) been coming down all week.

3. The town (is | was) in trouble, said the villager.
   Bandits (have | had) been scaring the townsfolk to death.

4. The reports (re | were) inaccurate, said the politician.
   Newspapers (have | had) been looking for a new scandal.

5. The company (is | was) in great peril, said the CEO.
   The shareholders (have | had) been discussing a hostile takeover.

6. The offense (needs | needed) to work harder, said the coach.
   The team (has | had) been lagging since halftime.

7. The market (is | was) up today, said the broker.
   The economy (has | had) been improving all week long.

8. The animals (are | were) frightened, thought the zookeeper.
   The monkeys (have | had) been shaking their cage all afternoon.

9. The city (is | was) under siege, thought the judge.
   Violent crime had (has | had) been rising for nearly a decade.

10. The crew (is | was) acting suspicious, thought the first mate.
    The sailors (have | had) been meeting in secret at night.

11. It (is | was) cold in the workshop, thought the carpenter.
    The heat had (has | had) been broken for several weeks.

12. The books (are | were) covered in dust, thought the librarian.
    Nobody (has | had) been interested in them for decades.

13. Conditions in the field hospital (are | were) atrocious, thought the doctor.
    The patients (have | had) been getting worse all week.

14. The wedding guests (are | were) very kind, thought the bride.
    They (has | had) been giving sweet toasts all night.

15. The church (is | was) very blessed, thought the priest.
    The steeple (has | had) been spared from the terrible storm.

16. The new play (is | was) a great success, thought the actor.
    Opening night (has | had) been sold out for months already.
